# Provider Perspectives on Persistent Urinary Incontinence Following Obstetric Fistula Repair in Ethiopia

**DOI:** 10.3389/fgwh.2020.557224

**Published:** 2020-10-15

**Authors:** Laura E. Jacobson, Melaku Abriha Marye, Elena Phoutrides, Rahel Nardos

**Affiliations:** ^1^Oregon Health & Science University-Portland State University School of Public Health, Portland, OR, United States; ^2^Obstetrics and Gynecology, Hamlin Fistula Hospital, Mekelle, Ethiopia; ^3^Contra Costa Regional Medical Center, Martinez, CA, United States; ^4^Division of Female Pelvic Medicine and Reconstructive Surgery, Director of Global Health in OBGYN, Oregon Health & Science University, Portland, OR, United States; ^5^Kaiser Permanente, Portland, OR, United States

**Keywords:** obstetric fistula, Ethiopia, urinary incontinence, qualitative analysis, obstetrics & gynecology surgeries

## Abstract

Each year an estimated 50,000 to 100,000 women worldwide are affected by obstetric fistula. This devastating but preventable maternal morbidity leaves women incontinent, stigmatized, isolated, and often with a still birth. While fistula rates in Ethiopia have declined in recent years, estimates range from 7 to 40 percent of women suffer from persistent urinary incontinence after successful closure of their fistula. Few studies have focused on the unique experiences and challenges that providers face treating fistula patients, particularly those who experience persistent urinary incontinence. The goal of this research is to characterize the fistula provider's accounts of how to manage, support, and understand their patient's experience. Semi-structured interviews were conducted with a purposive sample of fistula care providers in Mekelle and Addis Ababa, Ethiopia. The main themes that emerged were a perceived exacerbated impact on quality of life for women with persistent urinary incontinence; a “double hit” of isolation from both their community and from other recovered fistula patients; how the church both influences how patients internalize their injury and provides them with hope and support; and the need for comprehensive and compassionate fistula care. Understanding how providers perceive and relate to their patients provides valuable insight to the unique challenges of treating this population and may better inform treatment programmes to address the gap between patient needs and current fistula care models.

## Introduction

Each year an estimated 50,000 to 100,000 women worldwide are affected by obstetric fistula, a devastating but preventable maternal morbidity that leaves a woman incontinent, stigmatized, isolated from their families and communities, and often mourning the death of their infant (1, 2). When a mother experiences obstructed or prolonged labor without timely access to emergency obstetric services, compression from the fetal head can cause ischemic injury to the bladder or bowel resulting in an obstetric fistula that leads to constant leaking of urine or feces (3). While surgical repair of this condition is largely successful in closing the fistula (4), a significant proportion of women with treated obstetric fistulas report persistent urinary incontinence even after surgical repair (5).

Ethiopia has responded to the United Nations Population Fund (UNFPA) global Campaign to End Fistula by establishing several initiatives to expand obstetric services offered at basic health facilities. This includes increasing the number of providers employed across the country and developing and maintaining health delivery infrastructure specific to fistula care (6, 7). Some estimates have the rates of obstetric fistula in Ethiopia as low as 0.6 per 1,000 in some areas of the country (8). However, a wide range from 7 to 40 percent of women suffer from persistent urinary incontinence after closure of their fistula (9, 10). This challenge, known as the “continence gap,” (11) is gaining attention as fistula rates in Ethiopia have declined over the past several years while other pelvic floor disorders and birth complications are on the rise (6, 12). Predictors of residual incontinence included age, number of years with fistula, prior attempts at fistula repair, and severity of the injury (13).

There is ample evidence in the literature to suggest that urinary and fecal incontinence from an obstetric fistula significantly impacts a woman's quality of life (3, 14, 15); however, very few studies have focused on the unique challenges that fistula providers face in treating and supporting the chronic needs of fistula patients, particularly those who experience persistent urinary incontinence. The goal of this research is to characterize fistula providers' accounts of how to understand their patients' experience and provide care and support for them.

## Methods

This qualitative study was conducted as part of a larger cross-sectional descriptive study (16) in Mekelle and Addis Ababa, Ethiopia, between 2016 and 2018 to characterize post-fistula repair incontinence. Semi-structured interviews were conducted by the first and second author with a purposive sample of health providers. Study participation criteria included providers involved in the direct care of fistula patients in a professional setting. Participants were excluded if they had no direct patient interaction such as administrative staff. Participants were recruited by snowball sampling from hospitals, fistulas treatment centers, and non-governmental organizations (NGO) in Mekelle and Addis Ababa, Ethiopia. Interviews took place in a quiet location at the place of the interviewee's employment such as an office or conference room. The semi-structured interview guide focused on topics related to the complexity of care for women with persistent urinary incontinence after successful fistula closure; supports and services needed for this population; and the overall context of health care in Ethiopia. This study was approved by the Institutional Review Boards at Oregon Health and Science University (Portland, Oregon, USA) and Mekelle University (Mekelle, Ethiopia). Participants provided written informed consent prior to participation.

### Qualitative Analysis

A sample of fistula care providers (*n* = 10), 60% male, age range 25–50 years, were enrolled in the study, including surgeons trained in fistula and incontinence surgery, general practice doctors, nurses, counselors, and social workers who worked at hospitals, fistula centers, and NGOs in Mekelle and Addis Ababa. The range of years of experiences for the providers in this samples was 2–20 years. Interviews ranged from 25 to 53 min in length. A thematic analysis was conducted with codes based deductively on initial interview questions and inductively on emergent themes (17) Interview transcripts were coded by the first author using Atlas.ti software version 8 and coding was reviewed by a second author for reliability.

## Results

Four main themes emerged from this analysis: a perceived exacerbated impact on quality of life (QOL) for women with persistent urinary incontinence; a “double hit” of isolation from both a patient's home community and from other recovered fistula patients; how the church both influences how patients internalize their injury and provides them with hope and support; and the need for comprehensive fistula care. The first theme, an exacerbated impact on QOL beyond typical fistula patients, emerged as respondents noted that when their patients received treatment for their fistula but remained incontinent, they were very distraught, frustrated, and confused. This was due to a lack of answer for why *their* surgery did not fully restore continence and disconcertment from seeing other women they encountered in the clinic or NGOs fully cured of their incontinence. One nurse who treated fistula patients displayed an understanding of how women with persistent urinary incontinence felt when she noted:

“*Yeah it is hard, they need more because already the fistula is repaired and she's still leaking. No child, no husband, even though the hole is closed, still leaking! She is neglected from the community. It is hard. Imagine. She is still leaking, and she asks why? Why? Already repaired? Why?” (Nurse)*

The next theme that emerged was a sense that incontinent patients felt isolation from both their community at home but also from the community of other fistula patients that they encountered at integration trainings and in clinics. This amounted to a “double hit” of isolation that had compounding effects beyond what a typical fistula patient would experience. One of the counselors who worked closely with recovered fistula patients as they trained to become advocates for safe motherhood stated that when the former patients stay at the center for training (usually a bonding experience for them), women who remain incontinent present with additional social challenges. He shared:

“*Some women that are not fully cured—there is a difficulty to go to all the trainings and social aspects. So they are not more engaged, even in their break time when they interact together. They talk about their families and things, but there are some with a difficulty that sit a little far away, and if they can knit, they do their knitting.” (Counselor)*

Additionally, a social worker acknowledged this same pattern of isolation within the community of former fistula patients when she noted:

“*For those who are fully cured, it's easy for them, they have nothing to care about. But for those who are not fully cured, sometimes there is difficulty and they are by themselves, even when they are [at the training]. The community is not supportive [at home] and even here they are alone. Sometimes they don't go to things, like if they are invited, they say no.” (Social worker)*

The third themes that emerged was the role of “the church,” which was the Orthodox Christian institution in this context. Providers acknowledged that the church was an important factor for these women both in internalizing their injury but also as a means to help support and give them hope. Providers indicated that many women with persistent urinary incontinence believed that it was because of the will of God or a “curse” that their surgery did not restore continence. Providers also acknowledged that because the church leaders are highly respected, they could play a role in dismantling these false narratives and help both the persistent urinary incontinence sufferers and the community members understand the causes of fistula and persistent urinary incontinence; encourage support of those who suffer; and promote treatment and prevention options. One nurse who would like to see the church take a more active role in fistula support added that:

“*The fistula is not only solved by the health professional. Everybody must be participating on that. The leaders, even the priests, they must know, because they can help the mothers by advising. It can happen to any women. The women are thinking they are bad persons, they are doing bad things and God will punish them.” (Nurse)*

Additionally, a counselor shared his perspective on the role that the church can play:

“*They feel it is because God became angry upon them, it is like a punishment from God. The priests they can help with this. To teach them the reality, the truth, the exact reason how they developed fistula. It is not because they are the cursed ones, it's not because they are the sinners, it's not because God wants to punish them, its only because of the prolonged labor at home or unskilled labor at health institutions.” (Counselor)*

The last theme that emerged consistently across the interviews was a need for comprehensive care that addresses the ongoing clinical need of women with persistent incontinence but also includes social support, psychological care, physical therapy, social reintegration, life skills, and spiritual counseling. Additionally, providers stressed that care needs to be compassionate given the unique and sensitive nature of the service delivery. A surgeon who cares for fistula patients remarked:

“*So I want to emphasize there is no singular answer to urinary incontinence after fistula surgery. And you have to individualize the treatment and you need to really think of how to develop a range of services for these patients. And it's always a process of listening and learning.” (Surgeon)*

Another surgeon reflected on delivering compassionate care from what he has learnt from treating this population:

“*The most important thing I've learnt is the “human” never ends, that is what I learnt. You may close the fistula successfully and when you close the fistula successfully, they may become dry comfortably, but then they ask you about their reproductive life and other things. Some are not dry. And you have to be patient, it's not like the others who you just tell what to do. You have to give them time, you have to give them eye contact, time for them to explain, laugh. So after that they will have trust in you. The [incontinence] problem is not easy, so you have to understand that.” (Surgeon)*

Finally, an experienced nurse shared her thoughts on the compassionate care delivery as a measure of quality for persistent urinary incontinence patients, stating:

“*Now this is the time to work on how health providers can become compassionate, caring, that's a training and an attitude that you develop from your teachers. So I think these are all policy issues. That's how I think the ministry of health should help. Investing in quality of care.” (Nurse)*

## Discussion

This analysis reveals insight and empathy from providers who are on the front lines of fistula treatment as they have learnt to understand the challenges faced by their patients. There is ample evidence that a positive and trusting relationship between the patient and provider is known to improve health outcomes in the United States and other developed countries (18–23). The Institute of Medicine's Framework of Health Systems Quality strives to ensure that care is safe, effective, timely, efficient, equitable, and person-centered (24), where person-centered care takes into account the preferences of the individual, the context of the setting, and the nature of the patient and provider relationship (25). While there is increasing focus on high-quality health systems to produce better outcomes with greater social value in developing countries (26), and efforts to characterize person-centered care for reproductive health equity world-wide (27) the literature on fistula care quality remains focused primarily on safety, effectiveness of surgery, and timeliness and not on person-centered concepts or long-term needs (2, 28). Further, person-centered care is particularly crucial for fistula patients who are already vulnerable due to the trauma they have endured, the social isolation they experience, and the global lack of gender equity that leaves them disenfranchised and unable advocate for themselves in healthcare settings (29, 30).

How providers understand and relate to their patients provides valuable insight into the unique challenges of treating women with fistula and persistent urinary incontinence and may better inform treatment programmes to provide more robust and person-centered care. These data can inform and enhance comprehensive care team design and approaches employed by the Fellowship Program in Female Pelvic Medicine and Reconstructive Surgery (FPMRS) in Ethiopia. This program, primarily initiated by fistula surgeons, and developed through multiple partnerships, is one of the first formal training programmes in FPMRS (known as urogynecology) in Ethiopia and is structured to provide high-quality care and to strengthen the health system in its local context (31). Further, NGOs focused on reintegration of fistula patients following repair may enhance programmes for those who suffer from persistent urinary incontinence by adding additional rehabilitation services, mental health, and spiritual counseling; assistance with hygiene management; economic reintegration guidance; as well as family planning and fertility care. Finally, religious institutions are very important in the daily lives of the majority of Ethiopians where 44% are Orthodox Christian and 34% are Muslim (32, 33). While the respondents in the study referenced Orthodox Christianity when they spoke of “the Church,” these findings can be extended more broadly to include other religious institutions. Health system and policy leaders may consider collaborating with diverse religious officials to promote fistula and persistent urinary incontinence awareness efforts to dismantle stigma and educate their constituents on the causes of fistula, strategies for prevention, and ways to support those who are suffering.

This study has limitations to acknowledge. The sample size of providers interviewed is small; however, the highly specific and specialized nature of the population justifies elevating the perspectives of these participants as they are on the frontlines of care for this population. Next, this study is cross sectional and does not take into account the shifting nature of fistula care in Ethiopia that is occurring over time (6). Further, this work does not include the perspectives of patients as logistical constraints prevented us from enrolling a sample of patients into the qualitative portion of this study. Since this was a sub-study, the vast majority of our staffing and funding went to the overarching clinical research study looking at the impact of persistent urinary incontinence on quality of life on fistula patients (16). We were unable to hire an additional coordinator to conduct patient interviews. This limits the insight from the patient-provider relationship as these data represent only one side. The perspective of the patients is highly valued and future research with patient participants is needed to expand on these findings and include the perspectives of patients.

## Conclusion

In conclusion, this study showed that persistent urinary incontinence is acknowledged and perceived by providers as uniquely challenging beyond the care for fistula. Understanding how providers perceive and relate to their patients provides valuable insight to the unique challenges of treating this population and may better inform treatment programmes and address the gap in patient experience in fistula care. By acknowledging the exacerbated impact on QOL, the double hit of isolation from both the community and from other recovered fistula patients, the role of the church, and the need for compassionate comprehensive fistula care, policymakers and program developers can further enhance their efforts to support women with fistula and persistent urinary incontinence in Ethiopia to improve the quality of the care delivered and the overall strength of the health system.

## Data Availability Statement

The raw data supporting the conclusions of this article will be made available by the authors, without undue reservation.

## Ethics Statement

The studies involving human participants were reviewed and approved by the Institutional Review Boards at Oregon Health and Science University (Portland, Oregon USA) and Mekelle University (Mekelle, Ethiopia). The participants provided their written informed consent to participate in this study.

## Author Contributions

LJ: data collection, data management, data analysis, and manuscript writing. MM: study design, supervision, and manuscript editing and review. EP: data collection and manuscript editing and review. RN: study design, supervision, and manuscript editing and review. All authors contributed to the article and approved the submitted version.

## Conflict of Interest

The authors declare that the research was conducted in the absence of any commercial or financial relationships that could be construed as a potential conflict of interest.
