# Supplementary material for: Provider Perspectives on Persistent Urinary Incontinence Following Obstetric Fistula Repair in Ethiopia
Source: Front Glob Womens Health. 2020 Oct 15;1:557224. doi: 10.3389/fgwh.2020.557224 (PMC8593944; doi:10.3389/fgwh.2020.557224)
Supplement: Supplementary file 1 [file Table_1.DOCX]

Post Repair Incontinence Study

In-depth Interview Guiding Questions for Experts in Fistula Care and Treatment

- Please describe your background and your experience with caring for women with fistula.
  - How long have you been working with fistula? What drew you to the field?
  - In your opinion, how has the field changed in the time since you have been working with fistula patients?
- What services are needed for women after they have surgery to repair fistula?
- What services are available for women after their fistula is repaired?
- In your experience, are there patients who need more support than others?
  - If so, why do you think this is the case?
  - What are some of the characteristics of women who need additional support?
- Describe a little bit about the process of reintegration for women after fistula repair.
  - In what circumstances to women have trouble with reintegration?
  - How do you counsel women who have challenges with reintegration?
- In your opinion, how large of a problem is post-repair incontinence?
- What options are currently available for a woman with incontinence after fistula repair?
- Probe: How do you counsel women who continue to have incontinence after fistula repair?
- What challenges do you experience with post-repair incontinence?
- What do you think needs to be done to address post-repair incontinence?
  - Resources? Outreach? Education? Rehabilitation?
- What is your experience with follow-up care with women treated for fistula?
  - In the settings you work, are women asked to return for follow-up care?
  - If so, do they normally return?
    - If not, why do you think they do not return?
    - What are the greatest challenges for women to access care for post-repair incontinence?
- In your opinion, what is the impact of post-repair incontinence on women’s quality of life?
- What is your opinions of the impact of the fellowship training on their ability to care for women with prolapse and incontinence?
- How has the fellowship changed your practice and how do you see yourself playing a role in changing how these women are cared for in the country going forward?
- What needs to happen overall in the country?
